# Thermal and Mechanical Properties of the Recycled and Virgin PET—Part I

**DOI:** 10.3390/polym14071326

**Published:** 2022-03-24

**Authors:** Yasemin Celik, Madina Shamsuyeva, Hans Josef Endres

**Affiliations:** IKK—Institute of Plastics and Circular Economy, Leibniz University Hanover, An der Universitaet 2, 30823 Garbsen, Germany; celik@ikk.uni-hannover.de (Y.C.); endres@ikk.uni-hannover.de (H.J.E.)

**Keywords:** polyethylene terephthalate (PET), plastic recyclates, plastic recycling, circular economy, mechanical recycling, evidence of recycling

## Abstract

In various countries, polyethylene terephthalate (PET) represents one of the plastics with a very high recycling rate. Since currently there is no analytical method enabling direct distinction between recycled PET (rPET) and virgin PET (vPET), there are various attempts to differentiate these materials indirectly. One of these approaches claims that the recycling of PET leads to polymer chain degradation, which is reflected in changed thermal, mechanical and crystalline properties, and testing of these properties can therefore be used to distinguish rPET and vPET. However, there are many sources leading to changes in the molecular structure and consequently to the changes of the above-mentioned properties of the PET. The purpose of this study is to analyze the glass transition and melting temperature, degree of crystallinity as well as bending and impact properties of 20 different commercially available PET recyclates from 14 suppliers and evaluate the results with respect to the literature values for vPET. The main results of this study show that the range of vPET properties is so broad that all of the corresponding properties of the tested rPET lie within this range.

## 1. Introduction

Polyethylene terephthalate (PET) is one of the most commonly used plastics worldwide both for food-grade applications such as bottles for water, soft drinks or juice or non-food applications such as packaging for cleaners or cosmetic as well as fibers for various textile products [1,2]. Besides this, in many countries, PET articles are coded with the resin identification code “1” and have a high recycling rate [2,3]. This is achieved by effective regional waste collection systems and corresponding recycling technologies. Recycled PET (rPET) is used to manufacture products such as containers, sleeping bag insulation, polyester fabrics, carpets or new PET bottles. According to the market forecast, the worldwide recycled PET market size was USD 8.9 billion in 2021 and is expected to increase to USD 11.7 billion by 2026 [4]. There are various regional legal and standardization approaches promoting both the recycling of plastic waste as well as the use of plastic recyclates in the production of plastic articles [5,6,7]. For examples, starting from 2025 in the European Union (EU), beverage bottles that are manufactured mainly from the PET must contain at least 25% of recycled plastic and starting from 2030 at least 30% [8]. Furthermore, the EU members have to ensure separate collection for recycling for 90% of single-use plastic products by 2029 [8]. Currently, 57% of the commercially available rPET is suitable for food applications such as bottle-to-bottle recycling. At the same time, about 40% of the rPET in the EU is downcycled to lower quality non-food-grade grades [9]. The food-grade quality of rPET is ensured by a certification of the purification efficiency of the used recycling plants by a so-called “challenge test” [10] performed by corresponding regional agencies such as the U.S. Food and Drug Administration (FDA) or European Food Safety Authority (EFSA) [7,11]. Although both mechanical and chemical approaches can be successfully used for the recycling of PET [12,13,14,15,16,17,18,19,20], currently, mainly mechanically recycled rPET is available commercially. During mechanical recycling, post-consumer PET is subjected to various pre-treatment steps such as sorting, crushing, washing and grinding to flakes to ensure the removal of contaminants such as metals, other plastics, paper, labels, wood, etc. Purification of the PET input stream i.e., the waste portion suitable for recycling, plays a crucial role for the recyclate quality. Particularly, the presence of contaminants such as polyvinyl chloride (PVC), polystyrene (PS), ethylene vinyl alcohol (EVOH) or polypropylene (PP) disturbs the further processing steps and reduces the material quality of the rPET [18]. Subsequently, the flakes are extensively dried and extruded using a vacuum degassing and a melt filtration system. Later, the resulting regranulates are subjected to a so-called solid-state polymerization (SSP) or solid-state postcondensation to increase molecular weight and intrinsic viscosity (IV) of the rPET [15]. During this process, the rPET flakes are treated at a temperature >200 °C under inert gas or a vacuum for a certain period of time, where esterification and transesterification reactions take place [12]. SSP is one of the recycling steps, which is very sensitive to the presence of the above-mentioned polymer contaminants. The influence of various SSP process parameters on properties of both virgin PET (vPET) and rPET has been extensively described in the literature [12,13,14,15,16,17,18,19,20,21]. The increase of the molecular weight of PET is reflected in the increased IV, melting point, crystallinity and mechanical properties. 

Generally, due to the additional manufacturing and transport expenses, high-quality plastic recyclates are more expensive than the virgin plastics of the same type. In addition, their availability is often limited due to long-term contracts, lack of suppliers or accessibility of input stream in a reproducible quality. Together with the above-mentioned legal regulations promoting the use of plastic recyclates, there is a growing demand for transparency regarding traceability of the recycled content. Currently, the recycled material content in plastic products is defined by corresponding certification agencies using, for example, a mass balance approach [5,22]. Furthermore, there are regional standards suggesting procedures for calculation of the recycled content such as DIN SPEC 91446 or DIN EN 15343 [6,23]. However, there is no analytical method to distinguish vPET and rPET, especially after the SSP step. The molecular weight (M_w_) of rPET can be increased after SSP from a range of 39,000–47,000 g/mol to approx. 70,000 g/mol [18] or from 20,000 g/mol to 60,000 g/mol [15]. At the same time, the exact values strongly depend on the processing conditions.

However, a widespread opinion in industry is that a distinction between rPET and vPET can be made indirectly by testing the material properties of the rPET and comparing them with the properties of vPET. However, there is no scientific study investigating this approach. In order to fill this information gap and eradicate this misunderstanding, a comprehensive study on the evaluation of rPET and vPET properties has to be performed. Even if a change in molecular structures or material properties can be observed due to repeated thermomechanical stress occurring during mechanical recycling, this approach is still incorrect, since there are various further causes resulting in the same effect such as insufficient pre-drying or stabilization as well as the use of unfavorable processing parameters.

The aim of the first part of this study is to evaluate the thermal properties, crystallinity, bending and impact properties of various commercially available rPET flakes and regranulates in comparison with vPET. This part focuses on the application-oriented characterization of various samples treated under constant conditions. The second part of this study will compare the effect of variable processing conditions on morphology, molecular structure and crystallinity of rPET and vPET.

## 2. Materials and Methods

### 2.1. Materials 

Post-consumer PET recyclates in the form of regranulates and flakes were used for testing. Various European recycling companies were asked for recycled PET samples of bottle quality. Regranulates have the same chemical composition as the input stream, i.e., flakes [5,6]. For this study, 20 clear, colorful or bluish samples, including 7 in the form of flakes and 13 in the form of granules, were supplied by 14 different manufacturers. The samples were provided without technical data sheets. Table 1 summarizes the sample nomenclature.

### 2.2. Methods

#### 2.2.1. Drying and Residual Moisture Measurement

Drying of the samples was conducted in the VD 115 vacuum drying oven from Binder GmbH (Holzgerlingen, Germany). According to the literature, the samples were dried at 120 °C for at least 5 h [24,25,26]. All of the samples were dried under the same conditions to ensure comparability of the data. The measurement of the residual moisture was performed at 160 °C with a sample weight of approx. 5 g using the AQUATRAC Station from Brabender Messtechnik GmbH & Co. KG (Duisburg, Germany) until a constant value was recorded.

#### 2.2.2. Extrusion

The flake samples were regranulated in the laboratory using a laboratory twin-screw extruder (11 mm) Process 11 from Thermo Fisher Scientific (Karlsruhe, Germany). The temperature of the feed zone was set at 260 °C and that of the plasticizing to the discharge zones at 275 °C.

#### 2.2.3. Injection Moulding

The injection moulding of all samples including both regranulated flakes and commercial granules was performed using BOY XS from Dr. Boy GmbH & Co. KG (Neustadt, Germany) at 295 °C under 500 bar pressure and a holding pressure time of 5 s. Test specimens Type B, according to the ISO 20753, were manufactured for the testing of bending and impact properties.

#### 2.2.4. Bending Testing 

Prior to bending testing, the specimens were conditioned at 23 °C and 50 r.H. for 88 h. A three-point bending test was conducted with the material testing machine Zwickiline 2.5 TH from ZwickRoell GmbH & Co. KG (Ulm, Germany) according to the ISO 178. Flexural modulus, flexural strength and elongation at strain were defined by testing five specimens for each sample. The test was conducted at a preload of 0.1 MPa and a speed of 2 mm/min.

#### 2.2.5. Impact Strength Testing

Prior to impact strength testing, the specimens were conditioned at 23 °C and 50 r.H. for 88 h. The instrumented Charpy notched impact strength test was carried out according to ISO 179 using ZR HIT25P (5 Joule) from Zwick-Roell GmbH & Co. KG (Ulm, Germany) at 23 °C. For the determination of the impact strength value, 10 specimens were tested for each sample.

#### 2.2.6. Thermal Properties and Crystallinity

The melting temperature and crystallinity of PET was determined by differential scanning calorimetry (DSC) in double determination. The test was performed according to ISO 11357 using DSC 214 Polyma from Netzsch GmbH & Co. KG (Selb, Germany) at a heating rate of 10.00 K/min up to 290 °C. The weight of each measured specimen was about 8 mg.

Furthermore, a cascade drying experiment with one commercial regranulate and one regranulated flake sample was conducted at 130 °C, 140 °C, 150 °C and 160 °C with a dwell time of 1.5 h under vacuum (2 mbar). These samples were subjected to DSC measurement for the analysis of melting behavior.

**Figure 1:**
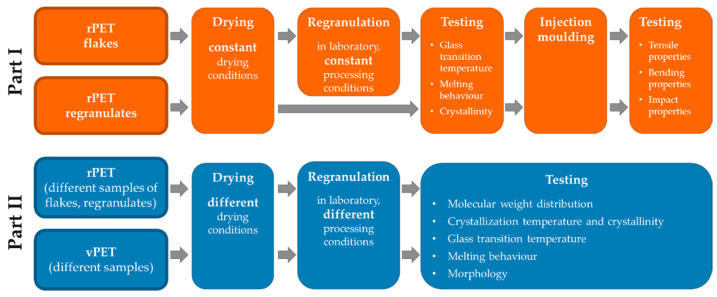
Summary of the materials and methods for the first and the second part of this study.

Figure 1 represents the overall procedure including materials, processing and testing methods used in the first and the second parts of this study.

#### 2.2.7. Statistical Proof of Experimental Results 

The ANOVA variance analysis was conducted using the Data Analysis tool of Microsoft Excel to prove significance of the test results obtained for flakes regranulated in the lab and commercially available regranulates. The *α*-value was set as 5% (0.05). For a significant result, *p*-value has to be ≤α-value. Additionally, the test value (F) needs to be higher than critical test value (F_crit_). A measurable significance means for a single factor ANOVA that at least some of the mean values are significantly different.

## 3. Results and Discussion

### 3.1. Moisture

**Figure 2:**
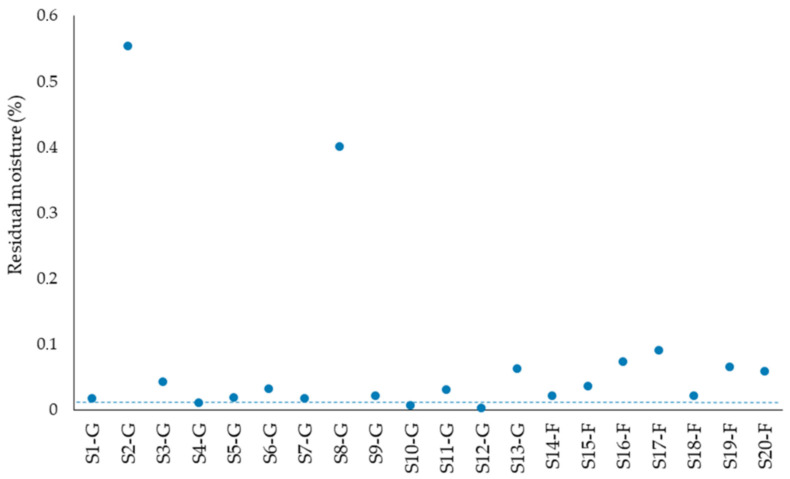
Residual moisture of rPET samples. The dotted line specifies literature value for the residual moisture.

PET is very sensitive to moisture, leading to hydrolysis of amorphous regions during processing at high temperatures, reduction in molecular weight and change of properties [27]. In the case of rPET, most of the moisture uptake takes place during the washing process, which is one of the integral pre-treatment steps during post-consumer plastic waste recycling. Consequently, drying of the rPET is also one of the most important steps, enabling high recyclate quality. The residual moisture values of the rPET samples dried at 120 °C for at least 5 h are represented in the Figure 2.

During drying of PET, it is important to consider that the acceptable residual moisture content is individual for various products depending on the further processing steps, such as for PET fibers, where a residual moisture of 0.03% is sufficient [12,28]. In film extrusion, due to a high degassing (approx. 20 mbar), it is sufficient to reduce only the surface moisture [29]. For the manufacture of molded parts from PET, the residual moisture should be less than 0.02% [12,30]. This value is specified in Figure 2 with a dotted line. The results show that under the selected laboratory conditions only few of the samples could be dried to the level suggested in the literature. At the same time, there is no unique approach for the drying of PET, and diverse conditions are reported in the literature (Table 2). The effectiveness of the drying is besides other factors dependent on crystallinity.

### 3.2. Thermal Properties, Crystallinity and Drying

**Figure 3:**
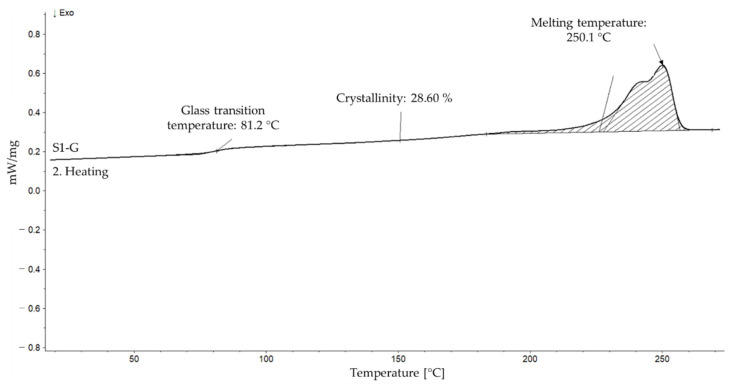
Exemplary evaluation of a DSC curve on the example of the S1-G sample.

Second heating curves during DSC measurement were used to determine glass transition temperature (*T*_g_), melting temperature and degree of crystallinity. Figure 3 represents an example of a DSC curve with defined values.

**Figure 4:**
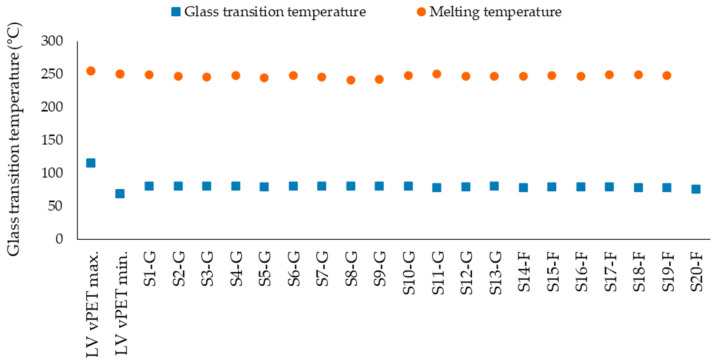
Thermal properties of rPET samples in comparison with literature values for vPET.

The DSC results of the tested samples and their comparison with the literature values are shown in the Figure 4. Comparison of the values shows that the rPET samples reach their melting point on average at about 243 °C. The lowest melting temperature is 241.1 °C (S8-G) and the highest 250.1 °C (S1-G). This is comparable with the literature values for vPET in the range of 250–255 °C [36]. Similar results are observed in the case of glass transition temperature. The values for rPET are between the minimum and maximum *T*_g_ values of the vPET, which are 69–115 °C [12]. The behavior of the *T*_g_ shows a very slight difference when commercial regranulates are compared with flakes; namely, the *T*_g_ is reached slightly earlier by the regranulated flake samples and lies in the range of 78.3–80.1 °C, while the *T*_g_ of the commercial regranulates is between 79.1–81.2 °C. There are different factors affecting *T*_g_ of polymers, such as molecular structure and weight distribution, polar groups or cross-linking degree. Among these factors, mainly molar mass is relevant for this study. An increase in molar mass leads to a decrease in polymer chain end concentration, leading to the reduction of their free volume and increase in *T*_g_. Consequently, the regranulated flakes have probably lower molar mass than industrial regranulates. This behavior can be a result of higher moisture content. Therefore, pre-drying seems to have a higher impact on *T*_g_ than whether it is a vPET or rPET. 

**Figure 5:**
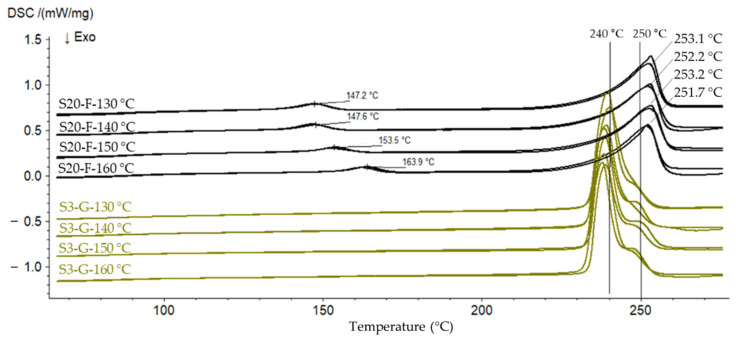
Melting curves of commercially granulated rPET (S3-G) and laboratory granulated rPET flakes (S20-F) dried at different temperatures from 130–160 °C.

To check this assumption, two randomly selected samples (one commercial regranulate and one laboratory-regranulated flake sample) are subjected to a cascade drying at 130 °C, 140 °C, 150 °C and 160 °C under 2 mbar vacuum with a dwell time of 1.5 h. The samples extracted after each drying step are subjected to the DSC analysis. Figure 5 represents the comparison of the melting behavior of the rPET samples dried at the various temperatures. Regardless of the drying temperature, a melting temperature of 240 °C on average with a peak shoulder at about 250 °C is observed for the commercial regranulates, while the regranulated flakes have a slightly higher melting temperature of 252–253 °C with a less pronounced shoulder before the peak. The shoulders, including both area and position, reflect the thermal history of the samples [37,38]. Basically, in the case of isothermal crystallization of semicrystalline polymers such as PET, crystallization temperature determines the size and stability of the crystallites. Particularly, a lower crystallization temperature leads to less perfect crystallites and thus a lower melting temperature [39]. In the case of the granulated flake sample, an additional peak at 147.2–163.9 °C is observed. This peak shifts to a higher temperature if the sample is dried at a higher temperature. The reason for these peaks is a so-called pre-melting [39]. Before the actual melting point of the PET recyclates starts at about 250 °C, in the pre-melting region the melting starts beforehand. These peaks can be associated with the melting of smaller or imperfect crystals present on an amorphous region of samples [39,40,41], as reported for a peak at 130 °C observed by thermal treatment of PET fibers [39]. Since the commercial granulates do not show these pre-melting peaks, this can be a result of deficiencies in the drying and consequently a high moisture content as described above. Particularly, if the drying temperature and the corresponding peaks in Figure 5 are considered, it is noticeable that drying at a higher temperature shifts the peaks to higher temperatures, i.e., towards the basic melting range.

**Figure 6:**
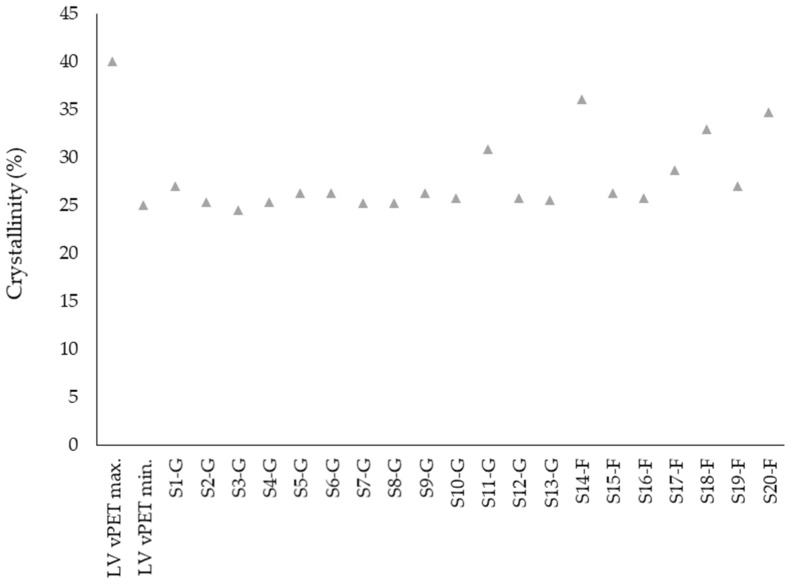
Crystallinity of rPET samples in comparison with literature values for vPET.

The material properties of a polymer such as transparency, density, ductility or strength are among further factors dependent on crystallinity of a given polymer. The DSC is one of the principal methods used to determine the crystallinity of a sample. The crystallinity of the tested samples is represented in Figure 6. The observed rPET results are comparable with the literature values of 29.7–32.9% for rPET [41]. Although the values for rPET are in line with those of vPET, in general they are closer to the minimal value of 25%. Regranulated flakes show a slightly higher crystallinity degree than the commercial regranulates, which is possibly also a result of the residual moisture content.

### 3.3. Bending Properties 

**Figure 7:**
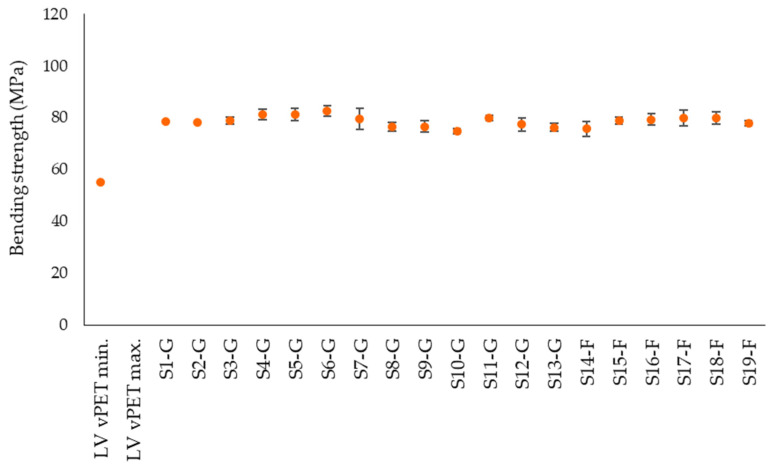
Experimentally determined bending strength of rPET samples in comparison with literature values for vPET.

**Figure 8:**
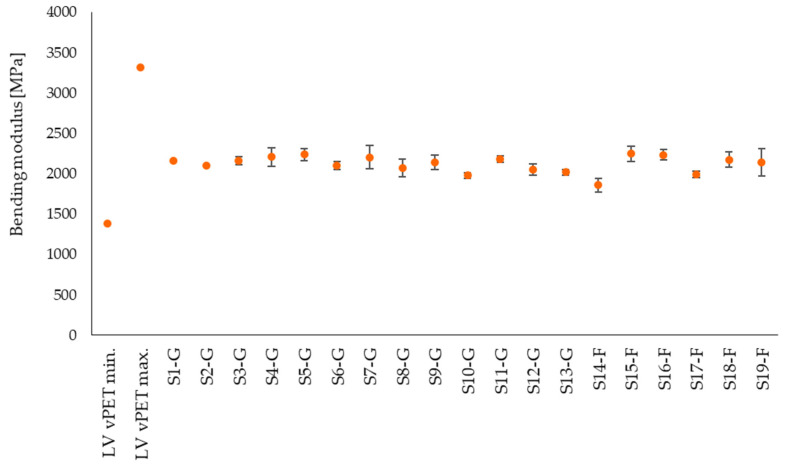
Experimentally determined bending modulus of rPET samples in comparison with literature values for vPET.

**Figure 9:**
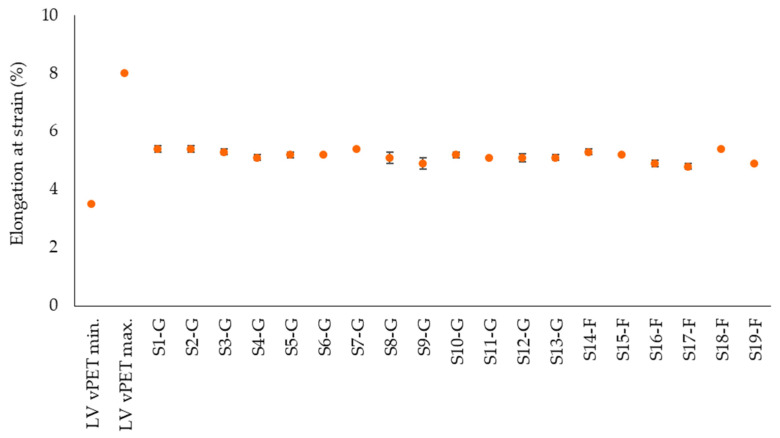
Experimentally determined elongation at flexural strength of rPET samples in comparison with literature values for vPET.

Figure 7, Figure 8 and Figure 9 represent bending strength, bending modulus and elongation at strain of the tested samples. Despite different moisture contents, the bending properties of the rPET are comparable with the literature values of vPET [42]. The bending strength of the flakes and regranulates is also similar (Figure 7). The flexural modulus values of the rPET samples are also comparable within the group but generally tend to be in the lower range of the literature values of vPET (Figure 8). The lower bending modulus means that the rPET samples are more flexible than vPET. The lowest modulus value of S14-F is 1860 MPa, which correlates with the highest crystallinity of 36.1% in Figure 6. However, the lowest crystallinity of 24.5% (S3-G) results in a modulus of 2162 MPa, which is not the highest value. This shows that further factors besides crystallinity need to be considered for an accurate evaluation of the results, such as molar mass or the presence of PET degradation products in the samples. The bending strain shows an average value at 5.2% (Figure 9). The lowest elongation of the commercial regranulates is 4.9% and of the granulated flake samples it is 4.8%, while the elongation of vPET ranges from 3.5–8%.

### 3.4. Impact Strength 

**Figure 10:**
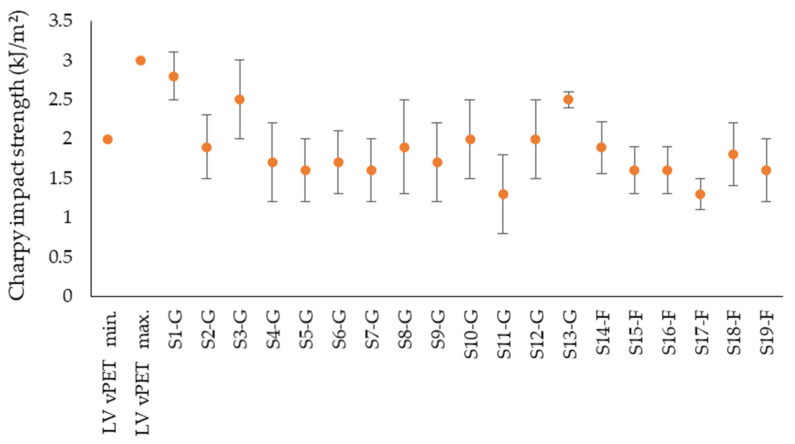
Impact strength of rPET samples in comparison with literature values for vPET.

Finally, Figure 10 represents the impact strength results of tested samples compared to literature values. A slight difference between the values of industrial regranulates and laboratory-regranulated flake samples can be observed. The commercial regranulates have an average value of 1.9 kJ/m^2^ in the range of 1.3–2.8 kJ/m^2^. This is comparable with the values for vPET in the range of 2–3 kJ/m^2^. In contrast, the regranulated flake samples are at an impact strength of 1.3–1.9 kJ/m^2^, with an average of 1.6 kJ/m^2^. The greater the impact energy consumed, the tougher the material. Accordingly, the industrial regranulates behave tougher in relation to the flake samples granulated in the laboratory. 

Based on the assumption that both crystallinity and molar mass influence toughness of a plastic, a higher crystallinity leads to a higher strength since intermolecular bonding is more significant in the crystalline phase. Sample S14-G has the highest crystallinity of 36.1% and an impact strength of only 1.89 kJ/m^2^. Similarly, the crystallinity of sample S11-G is 30.9% and impact strength 1.3 kJ/m^2^. The recognizably high error bars in Figure 10 can be explained by the reduction of the molar mass, since PET chains have suffered cracks during reprocessing PET [21,43]. Moreover, the heterogeneity of the polymer chains during the recycling process and presence of impurities promote this behavior [12]. Consequently, the simultaneous influence of molar weight distribution as well as presence of cleavage products on mechanical properties of at least three different samples of recycled and virgin treated under the same conditions should be investigated in the second part of this study.

### 3.5. Statistical Significance of the Obtained Results 

An ANOVA variance analysis was conducted to statistically evaluate whether there is a difference between the results obtained for flakes regranulated in the lab and commercially available regranulates. For a significant result, the *p*-value has to be ≤α-value. Furthermore, the test value F needs to be >F_crit_ (critical test value). A measurable significance means for a single factor ANOVA that at least some of the mean values are significantly different. The results represented in Table 3 show that there is a significant statistical difference between flakes and regranulates only in the case of the *T*_g_ and degree of crystallinity, and there is no significant difference in terms of the other properties. 

## 4. Conclusions

In the scope of this study, commercially available PET regranulates and flakes regranulated in a laboratory were tested in terms of their crystallinity, glass transition and melting temperature, as well as mechanical properties. The main results of this study show that the virgin and recycled PET are available in a very broad range of material properties, so that it is not possible to distinguish between virgin and recycled type based on its material properties.

At the same time, there is a demand for a more extensive review about the influence of processing steps on the molecular weight distribution and crystallinity of the virgin and recycled PET. This topic will be investigated in the second part of this study.

## Figures and Tables

**Table 1:** Description and nomenclature of samples.

Value/Sample	Nomenclature	Color
Minimal literature value for vPET	LV vPET min.	-
Maximal literature value for vPET	LV vPET max.	-
rPET granules 1	S1-G	Bluish
rPET granules 2	S2-G	Colorless
rPET granules 3	S3-G	Bluish
rPET granules 4	S4-G	Colorless
rPET granules 5	S5-G	Bluish
rPET granules 6	S6-G	Colorless
rPET granules 7	S7-G	Bluish
rPET granules 8	S8-G	Bluish
rPET granules 9	S9-G	Bluish
rPET granules 10	S10-G	Bluish
rPET granules 11	S11-G	Colorless
rPET granules 11	S12-G	Bluish
rPET granules 11	S13-G	Bluish
rPET flakes 1	S14-F	Colorless
rPET flakes 2	S15-F	Colorless
rPET flakes 3	S16-F	Colorless
rPET flakes 4	S17-F	Colorful
rPET flakes 5	S18-F	Colorless
rPET flakes 6	S19-F	Colorless
rPET flakes 7	S20-F	Colorless

**Table 2:** Drying temperature of PET reported in various sources.

Sample	Drying Conditions	Reference
PET	110–120 °C/3–4 h	[25]
PET-based epoxy resin films	120 °C/1 h	[30]
PET granules	120 °C/5 h	[24]
PET pellets	120 °C/10 h	[31]
PET/HDPE blends	120 °C/24 h	[32]
PET flakes	120 °C/-	[33]
Dry blend of PET/PLA	140 °C/6 h	[34]
rPET flakes	140 °C/-	[20]
Cylindrical PET chips	160 °C/3 h	[35]
Post-consumer PET	140–170 °C/3–7 h	[12]
	170 °C/6 h	

**Table 3:** Statistical analysis of the obtained results for flakes and commercial regranulates.

Property	F	*p*-Value	F_crit_	Statistical Significance
Residual moisture (%)	0.16	0.69	4.49	no
*T*_g_	19.37	0.00	4.49	yes
Melting temperature (°C)	4.48	0.05	4.49	no
Crystallinity (%)	8.93	0.01	4.49	yes
Bending strength (MPa)	0.33	0.58	4.54	no
Bending modulus (MPa)	0.05	0.83	4.54	no
Elongation at strain (%)	3.47	0.08	4.54	no
Charpy (kJ/m^2^)	2.61	0.13	4.54	no

## Data Availability

The data presented in this study are available in the article.
